# Differential effects of androgens on coronary blood flow regulation and arteriolar diameter in intact and castrated swine

**DOI:** 10.1186/2042-6410-3-10

**Published:** 2012-05-23

**Authors:** Erin K O’Connor, Jan R Ivey, Douglas K Bowles

**Affiliations:** 1 Department of Biomedical Sciences, University of Missouri, Columbia, MO, 65211, USA; 2 Dalton Cardiovascular Research Center, University of Missouri, Columbia, MO, 65211, USA

**Keywords:** Androgens, Coronary blood flow, Porcine, Vasomotor, Androgen receptor

## Abstract

**Background:**

Low endogenous testosterone levels have been shown to be a risk factor for the development of cardiovascular disease and cardiovascular benefits associated with testosterone replacement therapy are being advocated; however, the effects of endogenous testosterone levels on acute coronary vasomotor responses to androgen administration are not clear. The objective of this study was to compare the effects of acute androgen administration on *in vivo* coronary conductance and *in vitro* coronary microvascular diameter in intact and castrated male swine.

**Methods:**

Pigs received intracoronary infusions of physiologic levels (1–100 nM) of testosterone, the metabolite 5α-dihydrotestosterone, and the epimer epitestosterone while left anterior descending coronary blood flow and mean arterial pressure were continuously monitored. Following sacrifice, coronary arterioles were isolated, cannulated, and exposed to physiologic concentrations (1–100 nM) of testosterone, 5α-dihydrotestosterone, and epitestosterone. To evaluate effects of the androgen receptor on acute androgen dilation responses, real-time PCR and immunohistochemistry for androgen receptor were performed on conduit and resistance coronary vessels.

**Results:**

*In vivo*, testosterone and 5α-dihydrotestosterone produced greater increases in coronary conductance in the intact compared to the castrated males. *In vitro*, percent maximal dilation of microvessels was similar between intact and castrated males for testosterone and 5α-dihydrotestosterone. In both studies epitestosterone produced significant increases in conductance and microvessel diameter from baseline in the intact males. Androgen receptor mRNA expression and immunohistochemical staining were similar in intact and castrated males.

**Conclusions:**

Acute coronary vascular responses to exogenous androgen administration are increased by endogenous testosterone, an effect unrelated to changes in androgen receptor expression.

## Background

Cardiovascular disease is a leading cause of morbidity and mortality worldwide. For years the high incidence of cardiovascular disease in males was hypothesized to be related to the detrimental effects of testosterone on the vasculature. However, recent studies have demonstrated low testosterone levels are associated with a number of cardiovascular risk factors including obesity, hypertension, hypercholesterolemia, and hypertriglyceridemia [1,2]. Low endogenous testosterone levels have been linked to reduced exercise capacity in men with heart failure [3], carotid atherosclerosis [4], impaired vascular reactivity [5] and increased severity of coronary artery disease [6]. A similar relationship between endogenous androgen levels and cardiovascular disease has also been demonstrated in animal models. Ten weeks following the castration of male Sprague Dawley rats, aortic endothelial damage was detectable by electron microscopy that was absent in the intact males. Similar ultrastructural changes were noted in intact males treated with a 5α –reductase inhibitor [7]. Previous work from our lab demonstrated that in a swine model of post-angioplasty restenosis, neointima formation following coronary balloon injury was greater in castrated males compared to both intact males and hormone replaced castrated males [8].

As a result of these studies, androgen therapy in hypogonadal men for the purpose of cardioprotection is a concept that has gained popularity in recent years. Human studies have shown that men with coronary heart disease show improved myocardial perfusion and reduced arterial stiffness following chronic oral testosterone therapy [9]. Similarly, transdermal testosterone therapy reduced arterial stiffness in elderly hypogonadal men [10]. In a rabbit model of atherosclerosis, castrated male rabbits on a high cholesterol diet that received subcutaneous dihydrotestosterone supplementation demonstrated reduced aortic plaque area compared to the placebo group [11]. Acutely, testosterone and the non-aromatizable metabolite dihydrotestosterone have been identified as dilators of blood vessels in both human and animal studies. High dose intravenous testosterone increased flow mediated dilation in men with coronary artery disease [12]. Similarly, acute intracoronary administration of physiologic doses of testosterone increased vessel diameter and blood flow of the coronary vessels in men with coronary artery disease [13]. Various animal models using rodents, rabbits, dogs, and swine have also demonstrated an acute dilation to androgen in both *in vivo* and *in vitro* studies [14-18]. However, while the acute and chronic effects of exogenous androgen have been evaluated, it is unknown what role endogenous testosterone levels have on these vascular responses in humans or animals.

Previous studies on the cardiovascular effects of androgen have focused largely on the effects of testosterone and its non-aromatizable metabolites 5α and 5β-dihydrotestosterone, but to date the vascular responses of the epimer epitestosterone have not been evaluated. Epitestosterone is a naturally occurring androgen produced by the testis and adrenal gland in humans. It is structurally similar to testosterone but in humans it has not been shown to be a testosterone derivative [19]. Because of the constant ratio of testosterone to epitestosterone in the circulation, the majority of research on epitestosterone is related to its use as a marker of blood doping in athletes [20]. Several studies have also described epitestosterone as having weak anti-androgen effects based on its ability to inhibit testosterone conversion to dihydrotestosterone [21]. The majority of the current literature on epitestosterone indicates that it lacks any significant biologic activity [19], though several sources suggest it may contribute to the regulation of androgen dependent events [20,21]. Because of the structural similarity to testosterone and affinity for the androgen receptor, we hypothesized that epitestosterone would demonstrate similar vasomotor responses to testosterone and 5α-dihydrotestosterone when administered acutely.

Testosterone has been described as both a calcium channel antagonist [16,22-24] and potassium channel agonist [14,15,18]. More recently the androgen receptor has been identified as a possible key factor in the mechanisms of androgen induced vascular responses. Androgen’s stimulatory effects on angiogenesis [25] and inhibitory effects on plaque development in atherosclerosis [11,26] have been shown to be mediated at least in part to androgen signaling through the androgen receptor. In coronary arteries from 24 men, there was a negative correlation between androgen receptor mRNA expression in the media and plaque area [27]. Recently testosterone has been shown to upregulate the phosphorylation of eNOS and production of nitric oxide through activation of the androgen receptor in human aortic endothelial cells, providing a potential mechanism for the acute androgen induced vascular dilation demonstrated both *in vitro* and *in vivo*[28]. Given the association of the androgen receptor with the production of a critical mediator of vascular tone suggests that endogenous testosterone levels and subsequently, androgen receptor expression, may affect the acute dilation responses to exogenous androgen administration. If confirmed, the results could have important clinical implications for chronic therapy targeted at improving the vascular health of hypogonadal men. We therefore sought to evaluate the effects of endogenous androgen on the acute vasomotor responses to exogenous androgen administration in intact and castrated swine.

## Methods

### Animals

Male intact and castrated swine, ages ranging from 3 months to 5.5 years (intacts 20.83 ± 4.79 months; castrates 3.80 ± 0.39 months) and weights ranging from 25–100 kg (intacts 69.76 ± 5.89 kg; castrates 31.20 ± 1.59 kg) were obtained for use in these studies. Domestic swine from the University of Missouri were used for the majority of the studies (Columbia, MO, USA). Minnesota Mini (NSRRC:0005) and Yucatan (NSRRC:0012) males were obtained from the National Swine Resource and Research Center (www.nsrrc.missouri.edu) (Columbia, MO, USA). Additional Yucatans were obtained from Sinclair Research Farm (Columbia, MO, USA). All swine were housed at the University of Missouri research animal facilities and fed a standard pig chow (Purina Lab Mini-pig Breeder Diet 5082 or Purina Lab Mini-pig Grower Diet 5L80). Animal protocols were approved by the University of Missouri Animal Care and Use Committee in accordance with the Guide for the Care and Use of Laboratory Animals (National Academy Press, Washington, D.C., 1996).

### Flow studies

Pigs were sedated with a combination of Telazol (5 mg/kg) and xylazine (2.25 mg/kg) and maintained under isoflurane anesthesia (2–2.5%). A thoracotomy was performed at the 3^rd^-4^th^ intercostal space to gain access to the left anterior descending coronary artery (LAD). Following blunt dissection, a transonic perivascular flow probe (Transonic Systems Inc., Ithaca, NY, USA) was positioned at the proximal aspect of the LAD. A 6F guide catheter (Boston Scientific, Quincy, MA, USA) was then introduced through a 7F sheath placed in the right femoral artery and positioned at the left main coronary artery. An infusion catheter (Spectranetics, Colorado Springs, CO, USA) was passed through the guide catheter and positioned in the LAD just distal to the position of the flow probe. Placement of the flow probe and infusion catheter was confirmed using angiography.

In a subset of animals, a chronic intravascular LAD catheter was inserted in place of the femoral artery infusion catheter. An intravascular polyethylene aortic catheter was also placed to allow for pressure measurements. The perivascular flow probe was positioned as previously described. The catheters and flow probe were exteriorized through the intercostal spaces and tunneled subcutaneously to the dorsal cervical region. Pigs were recovered and flow studies performed in the conscious animals several days following surgery.

In both the anesthetized and conscious animals, androgens were infused into the LAD for 5 minute intervals. The androgens evaluated were testosterone, 5α-dihydrotestosterone, and epitestosterone (Sigma, St. Louis, MO, USA). 50 μM (14 μg/mL), 500 μM (140 μg/mL), and 5 mM (1400 μg/mL) solutions of androgen in 100% ethanol were diluted in saline to make 50, 500, and 5,000 nM stocks of androgen. The order of the androgens was randomly selected for each animal. Depending on the animal’s baseline coronary blood flow, the rate of infusion was altered to achieve final coronary androgen concentrations of 1, 10, and 100 nM during the 5 minute infusions. At the beginning of the study a vehicle control of 0.1% Ethanol was infused for 5 minutes. An adenosine bolus (5ug/kg) was also administered prior to androgen infusion to achieve maximal dilation of the coronary vessels. In between androgens, saline boluses were administered to wash out all remaining androgen and the animal’s coronary blood flow was allowed to stabilize prior to administration of the next androgen.

In a separate group of anesthetized intact animals, androgens were infused individually and in the presence of flutamide, an androgen receptor blocker. In these experiments, the 500 nM doses of 5α-dihydrotestosterone and epitestosterone were administered over 5 minutes with saline flushes in between each androgen. Subsequently, a 10 mM (2.76 mg/mL) solution of flutamide in 100% ethanol was diluted in saline to make a 500 μM solution and infused over 5 minutes at a rate to achieve coronary concentrations of 10 μM flutamide. Following the flutamide infusion, the 500 nM androgens and the 500 μM flutamide were infused simultaneously over 5 minute intervals, with saline flushes in between androgens. At the beginning of the study a vehicle control of 5% ethanol was infused for 5 minutes to account for the ethanol concentration in the flutamide stock.

Flow study data was collected using a Transonic flow meter (Transonic Systems Inc., Ithaca, NY, USA) and Chart 5 PowerLab Software (ADInstruments, Colorado Springs, CO, USA). Coronary blood flow and mean arterial pressure were measured continuously during the infusions. Upon completion of the flow study, the heart was removed from the anesthetized animal for subsequent *in vitro* studies.

### Microvessel studies

An ~3 cm diameter section of myocardium at the apex of the left ventricle was harvested from explanted hearts for microvessel studies. Vessels 100–300 μM in diameter were dissected from the harvested section of left ventricle. Vessels were then cannulated with glass micropipettes and pressurized to 60 cm H_2_O in physiologic salt solution (PSS) with albumin (NaCl 145 mM, KCl 4.7 mM, CaCl_2_·H_2_0 2.0 mM, MgSo_4_·7 H_2_0 1.17 mM, MOPS 3 mM, NaH_2_P0_4_ 1.2 mM, glucose 5 mM, pyruvate 2 mM, EDTA 0.02 mM, albumin 10 g, pH 7.4). Vessels were maintained at 37°C and washed 3–4 times with PSS at 15 minute intervals during an initial equilibration period. Vessels that did not achieve a basal tone of at least 20-40% during the equilibration period were pre-constricted with endothelin-1 (3 pM-10 nM). Vessels preconstricted with endothelin-1 were washed 2–3 times prior to androgen administration. Increasing concentrations of androgen were cumulatively administered directly into the bath for 5 minute intervals. The androgens evaluated were testosterone, 5α-dihydrotestosterone, and epitestosterone (Sigma, St. Louis, MO, USA). 10 mM (2800 μg/mL) stocks of androgen dissolved in 100% ethanol were diluted in PSS buffer to make 0.1 μM, 1.0 μM and 10 μM solutions of androgen. Final concentrations of androgen achieved in the bath were 1, 10 and 100 nM. The order of the androgens was randomly selected for each animal. Vessels were washed 2 times with PSS at 15 minute intervals in between androgens. If vessels did not achieve at least 20% tone following the washout period, endothelin-1 was administered as previously described. Following the androgen dose response experiments, the integrity of the endothelium was confirmed with 0.01 μM bradykinin in the bath. The experiments were terminated with a final wash of the vessel with a Ca free solution (NaCl 147 mM, KCl 4.7 mM, MgSo_4_·7 H_2_0 1.17 mM). Changes in microvessel diameter were recorded using Chart 5 PowerLab Software (ADInstruments, Colorado Springs, CO, USA).

### qRT-PCR

Quantitative RT- PCR (qRT-PCR) was performed to amplify the androgen receptor and the constitutively expressed gene, glyceraldehyde-3-phosphate dehydrogenase (GAPDH) as previously described with modifications [29,30]. LAD segments (10-mm axial length) and microvessels were quick-frozen in liquid nitrogen and stored at −80°C until processed. Frozen LAD segments were pulverized under liquid nitrogen, and total RNA was extracted (TRI Reagent, Molecular Research Center, Cincinnati, OH). RNA was extracted from microvessels using the PicoPure RNA Isolation kit (MDS Analytical Technologies, Sunnyville, CA). cDNA was transcribed from total RNA using the High Capacity cDNA Reverse Transcription Kit (Applied Biosystems, Foster City, CA) using 50 units of Multiscribe Reverse Transcriptase in a 20-μl reaction containing 50 μM random primers, 20 units of RNase Inhibitor, 100 mM dNTPs, and RT Buffer. Quantitative RT-PCR was performed on a MyiQ Single Color Real-Time PCR cycler (Bio-Rad, Hercules, CA). Each 25-μl reaction contained iQ SYBR Green Supermix (Bio-Rad, 170–8882), 0.8 μM forward and reverse primers, and 100 ng of cDNA. Each primer set was optimized for cycle time and annealing temperature. The PCR reactions were initiated by a 3 min incubation at 95°C followed by 50 cycles of 95°C (60 sec), 58°C (60 sec), and 72°C (60 sec) for androgen receptor. GAPDH was amplified using 40 cycles of 95°C (15 sec), 58°C (30 sec), and 72°C (30 sec). Primers were designed on the basis of published sequences from domestic porcine (Sus scrofa). The primer sets were as follows: androgen receptor: sense, 5’-GTC CCG AAT GTA CAG CCA GT-3’; antisense, 5’-CTT GAG CAG GAT GTG GGA TT-3’; GAPDH: sense, 5’-TCA AGA AGG TGG TGA AGC AG-3’; antisense, 5’-TGT CGT ACG AGG AAA TGA GC-3’. Target gene expression was normalized to GAPDH ribosomal RNA using the 2 ^-ΔΔCt^ method [31]. Linearity and efficiency of each PCR condition were verified by creating a standard curve plotting the critical threshold vs. log of the dilution of cDNA.

### Immunohistochemistry

Androgen receptor protein expression was evaluated in the intact and castrated males using immunohistochemistry with a rabbit polyclonal antibody directed against the androgen receptor as described previously [8,32]. Sections were incubated with avidin–biotin two-step blocking solution (Vector SP-2001) to inhibit background staining and in 3% hydrogen peroxide to inhibit endogenous peroxidase. Non-serum protein block (Dako X909) was applied to inhibit non-specific protein binding. Primary antibody for androgen receptor (1:200, PA1-110, Thermo Scientific) was incubated overnight at 4°C. After appropriate washing steps, sections were incubated with biotinylated secondary antibody in phosphate-buffered saline containing 15 mM sodium azide and peroxidase-labeled streptavidin (Dako LSAB + kit, peroxidase, K0690). Diaminobenzidine (DAB, Dako) was applied for 5 minutes to visualization of the reaction product. Sections were photographed with an Olympus BX40 photomicroscope and Spot Insight Colour camera (Diagnostic Instruments). One section of left anterior descending coronary artery and 2–3 microvessels for each animal were analyzed. Digital color (RGB) images were obtained. Quantification of the density of positive staining for androgen receptor in the intima and media was performed for each vessel utilizing ImagePro Plus (Media Cybernetics). Briefly, pixels within the region of interest identified as positive based on color selection were analyzed for mean pixel density and percent of area positive (area positive/total area).

### Statistical analysis

*In vivo* data is expressed as percent change in coronary conductance during each 5 minute infusion. Conductance was calculated from coronary blood flow and mean arterial pressure measurements taken at 2 second intervals during the flow studies. The baseline conductance used for analysis was the mean conductance over a 30 second period immediately prior to each infusion. The conductance for each infusion was the maximal mean conductance over a 30 second period during the 5 minute infusion. Due to a transient vehicle induced dilation observed at the beginning of the infusions, the first 80 seconds of each infusion was excluded from analysis. Microvessel data is expressed as percent maximal dilation. The maximal dilation of the coronary arteriole was the maximum diameter of the vessel achieved during the experiment. Percent maximal dilation was calculated using [(Da- Db)/(Dmax-Db) x 100], where Da is diameter after androgen, Db is baseline diameter, and Dmax is maximal diameter. Vessels that demonstrated less than 20% tone following endothelin-1 or less than 40% maximal dilation to bradykinin were excluded from analysis.

Statistical analyses were performed using SigmaStat software (Systat Software, Inc., San Jose, CA) and PASW Statistics 18 software (SPSS, Inc., Chicago, IL). Data are expressed as mean ± SEM. Group comparisons for *in vivo* and microvessel data were analyzed using 2-Way ANOVA with the Holm-Sidak post hoc test applied for significant interactions. The Kruskal Wallis ANOVA was used when data were not normally distributed. Individual androgen response comparisons between intacts and castrates were analyzed using t-tests and the Mann–Whitney Rank Sum test when data were not normally distributed. Individual androgen response comparisons to baseline for intacts and castrates were analyzed using paired t-tests or signed rank tests. Group comparisons for PCR and immunohistochemistry data were analyzed using t-tests or the Mann–Whitney Rank Sum test. A P value ≤ 0.05 was deemed significant.

## Results

### Flow studies in anesthetized swine

Baseline coronary blood flow (CBF) measured at the beginning of the flow studies prior to drug infusion was similar between intact and castrated males (33.46 ± 5.1 and 35.63 ± 4.94 mL/min, respectively; Table 1). During the androgen infusions, the intact males demonstrated a significant increase in CBF from baseline for the 1, 10, and 100 nM doses of testosterone (5.31 ± 1.90, 8.72 ± 2.90, and 6.72 ± 2.65%, respectively), the 1, 10, and 100 nM doses of 5α-dihydrotestosterone (12.17 ± 5.62, 3.51 ± 1.45, 6.94 ± 2.44%, respectively), and the 10 nM dose of epitestosterone (8.09 ± 3.71%). In contrast to the intacts, the castrated males demonstrated a significant increase in CBF only after the 100 nM dose of testosterone (2.86 ± 1.16%). The increases in blood flow during the 10 nM testosterone and 1 nM 5α-dihydrotestosterone infusions in the intacts were significantly greater than the responses present in the castrated males (8.72 ± 2.90 vs. 2.57 ± 1.62%, and 12.17 ± 5.62 vs. -1.64 ± 3.29%, respectively).

**Table 1:** ***In Vivo *Hemodynamic Responses**

	**Intacts**			**Castrates**		
Baseline CBF (mL/min)	33.46	±	5.10	35.63	±	4.94
% Δ Flow						
T 1 nM	5.31	±	1.90 *	−0.84	±	2.43
T 10 nM	8.72	±	2.90 *†	2.57	±	1.62
T 100 nM	6.72	±	2.65 *	2.86	±	1.16 *
DHT 1 nM	12.17	±	5.62 *†	−1.64	±	3.29
DHT 10 nM	3.51	±	1.45 *	1.54	±	2.14
DHT 100 nM	6.94	±	2.44 *	2.08	±	1.20
EpiT 1 nM	7.85	±	4.07	4.38	±	2.63
EpiT 10 nM	8.09	±	3.71 *	4.80	±	2.44
EpiT 100 nM	3.59	±	1.78	2.60	±	3.56
Baseline MAP (mmHg)	64.93	±	8.99	76.33	±	8.85
% Δ Pressure						
T 1 nM	−0.13	±	0.47	−0.23	±	0.38
T 10 nM	−0.51	±	0.38	−1.92	±	0.53 *
T 100 nM	−0.4	±	0.51	−1.64	±	0.49 *
DHT 1 nM	−0.78	±	0.52	−0.71	±	0.63
DHT 10 nM	−0.7	±	0.32	−1.12	±	0.74
DHT 100 nM	−0.33	±	0.26	−0.63	±	0.42
EpiT 1 nM	−0.37	±	0.53	−0.95	±	0.52
EpiT 10 nM	−0.38	±	0.33	−0.63	±	0.33
EpiT 100 nM	−0.64	±	0.40	−0.93	±	0.42 *

Coronary blood flow (CBF) and mean arterial pressure (MAP) responses to infusions of Testosterone (T), 5α-Dihydrotestosterone (DHT), and Epitestosterone (EpiT). *p < 0.05 vs. baseline, †p < 0.05 vs. castrates. n = 7-8 for intacts, n = 5-9 for castrates. Values are mean ± SEM.

Baseline mean arterial pressure (MAP) measured at the start of the flow studies was not significantly different between the intact and castrated males (64.93 ± 8.99 and 76.33 ± 8.85 mmHg, respectively; Table 1). During the androgen infusions, there was a small but significant drop in MAP from baseline in the castrates for the 10 and 100 nM doses of testosterone (−1.92 ± 0.53 and −1.64 ± 0.49%, respectively) and the 100 nM dose of epitestosterone (−0.93 ± 0.42%). MAP did not vary significantly from baseline during the intact androgen infusions.

**Figure 1:**
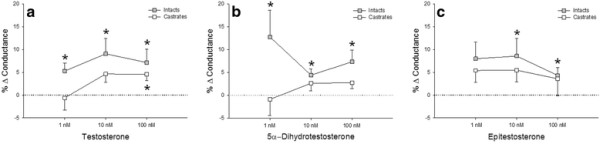
*** In vivo *effects of androgen on the coronary circulation in anesthetized swine.** Percent change in left anterior descending coronary conductance from baseline for intact and castrated males during infusions of (**a**) Testosterone, (**b**) 5α-Dihydrotestosterone and (**c**) Epitestosterone (n = 7-8 for intacts, n = 5-9 for castrates). Values are mean ± SEM. *p < 0.05 vs. baseline. The testosterone and 5α-dihydrotestosterone infusions demonstrate a main effect of castration on coronary conductance.

Coronary conductance was significantly increased from baseline in the intacts for the 1, 10, and 100 nM doses of testosterone (5.29 ± 1.70, 9.07 ± 3.39, and 7.14 ± 2.96%, respectively), the 1, 10, and 100 nM doses of 5α-dihydrotestosterone (12.76 ± 5.90, 4.40 ± 1.38, and 7.37 ± 2.53%, respectively), and the 10 and 100 nM doses of epitestosterone (8.57 ± 3.86 and 4.32 ± 1.74%, respectively; Figure 1). Conversely, conductance was only increased in the castrated males from baseline during the 100 nM dose of testosterone (4.56 ± 1.33%).

### Flow studies in conscious swine

**Figure 2:**
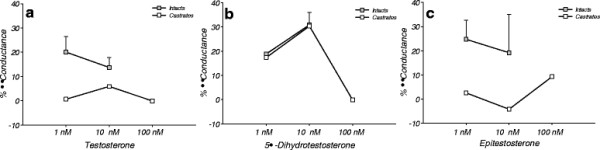
*** In vivo* effects of androgen on the coronary circulation in conscious swine.** Percent change in left anterior descending coronary conductance from baseline for intact, castrated, and testosterone replaced males during infusions of (**a**) Testosterone, (**b**) 5α-Dihydrotestosterone and (**c**) Epitestosterone (n = 2 for intact, n = 1 for castrates, n = 1-2 for hormone replaced). Values are mean ± SEM. The testosterone and epitestosterone infusions demonstrate a trend for increased coronary conductance in the intact males compared to the castrated and hormone replaced males.

The intact animals demonstrated increased coronary conductance during the 1 and 10 nM testosterone infusions (20.03 ± 6.45 vs. 0.61%, and 13.71 ± 4.04 vs. 5.89%, respectively), as well as the 1 and 10 nM epitestosterone infusions (24.73 ± 7.96 vs. 2.52%, and 19.18 ± 15.97 vs. -4.25%, respectively) compared to the castrated animals (Figure 2). Thus, these responses in conscious swine were similar to that observed in anesthetized animals. However, due to the small sample sizes, a statistical analysis was not performed.

### Microvessel studies

**Figure 3:**
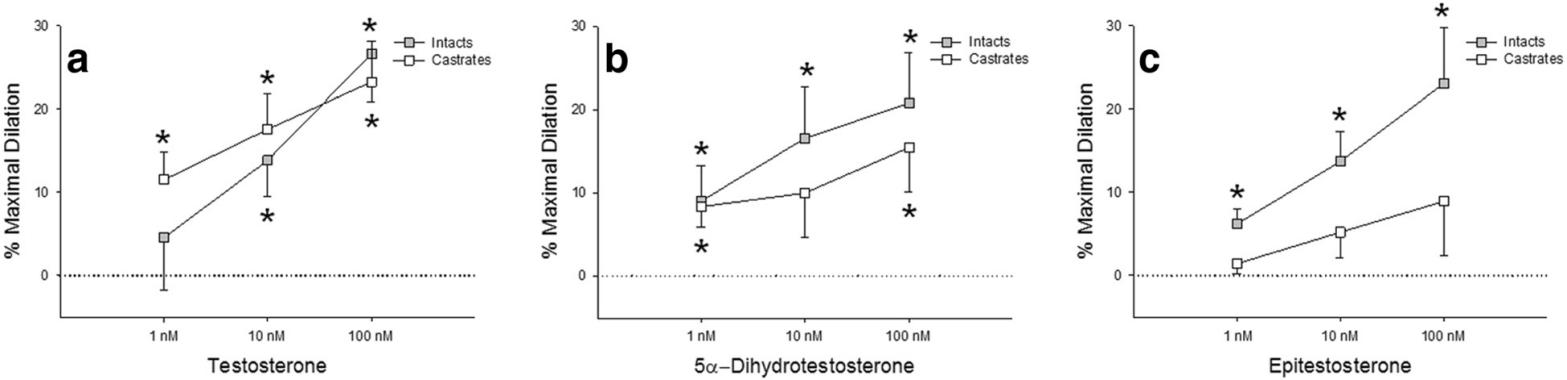
*** In vitro* effects of androgen on coronary microvessel diameter.** Percent maximal dilation of coronary resistance vessels from intact and castrated males exposed to physiologic concentrations of (**a**) Testosterone, (**b**) 5α-Dihydrotestosterone, and (**c**) Epitestosterone (n = 12 for intacts, n = 9 for castrates). Values are mean ± SEM. *p < 0.05 vs. baseline. The epitestosterone exposures demonstrate a main effect of castration on coronary microvessel diameter.

Coronary microvessels from intact males demonstrated a significant dilation from baseline to 10 and 100 nM testosterone (13.86 ± 4.39 and 26.63 ± 5.74% maximal dilation, respectively); 1, 10, and 100 nM concentrations of 5α-dihydrotestosterone (9.00 ± 4.29, 16.56 ± 6.15, and 20.76 ± 6.08% maximal dilation, respectively); and 1, 10, and 100 nM epitestosterone (6.19 ± 1.88, 13.73 ± 3.64, and 23.07 ± 6.67% maximal dilation, respectively; Figure 3). Arterioles from castrated males showed similar dilation responses to 1, 10, and 100 nM testosterone (11.54 ± 3.30, 17.63 ± 4.23, and 23.29 ± 4.87% maximal dilation, respectively) and 1 and 100 nM 5α-dihydrotestosterone (8.37 ± 2.52 and 15.44 ± 5.25% maximal dilation, respectively). However arterioles from castrated males did not show a significant dilation compared to baseline for epitestosterone (1.40 ± 1.24, 5.19 ± 3.09, 8.93 ± 6.50% maximal dilation).

### Flutamide experiments

**Figure 4:**
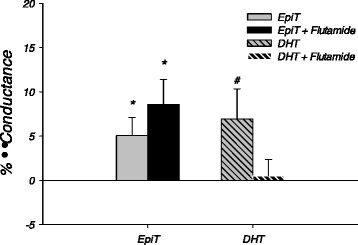
*** In vivo* effects of flutamide on coronary vascular responses to androgen.** Percent change in coronary conductance from baseline for intact males during 10 nM infusions of Epitestosterone (EpiT) and 5α-Dihydrotestosterone (DHT) alone and in the presence of 10 μM flutamide (n = 6). Values are mean ± SEM. *p < 0.05, # <0.06 vs. baseline.

Intact animals demonstrated a significant increase in coronary conductance from baseline during the epitestosterone infusion (5.05 ± 2.06%) that was not diminished by flutamide (Figure 4). Dihydrotestosterone infusion also produced an ~7% increase in coronary conductance in the absence of flutamide, while in the presence of flutamide dihydrotestosterone did not increase conductance over baseline (0.47 ± 1.90).

### qRT-PCR analysis

**Figure 5:**
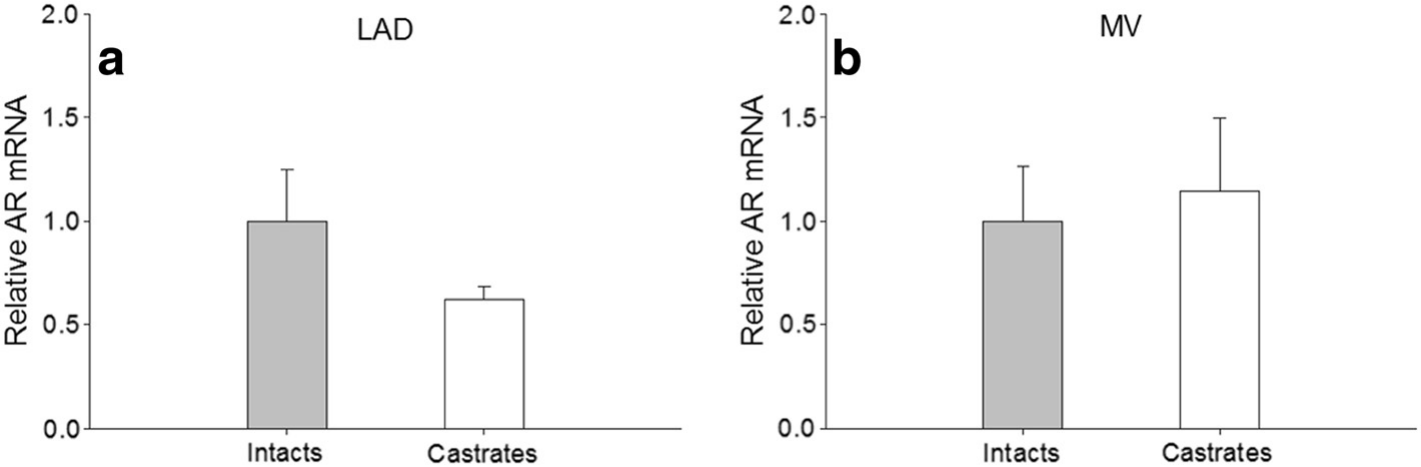
** Androgen receptor gene expression in intact and castrated males.** Quantitative RT-PCR analysis of androgen receptor (AR) mRNA levels in intact and castrated males in the (**a**) left anterior descending coronary artery (LAD) (n = 9 for intacts, n = 7 for castrates) and (**b**) left ventricular microvessels (MV) (n = 10 for intacts, n = 8 for castrates). AR gene expression was normalized to GAPDH gene expression using the 2 ^-ΔΔCT^ method. Values are mean ± SEM.

In the LAD, the castrated males showed a trend for reduced androgen receptor mRNA expression though the difference was not significantly different between the intacts and castrates (1.00 ± 0.25 and 0.62 ± 0.06, respectively) (Figure 5A). In coronary resistance vessels, androgen receptor mRNA levels were similar in intact and castrated males (1.00 ± 0.27 and 1.15 ±0.35, respectively) (Figure 5B).

### Immunohistochemistry

**Figure 6:**
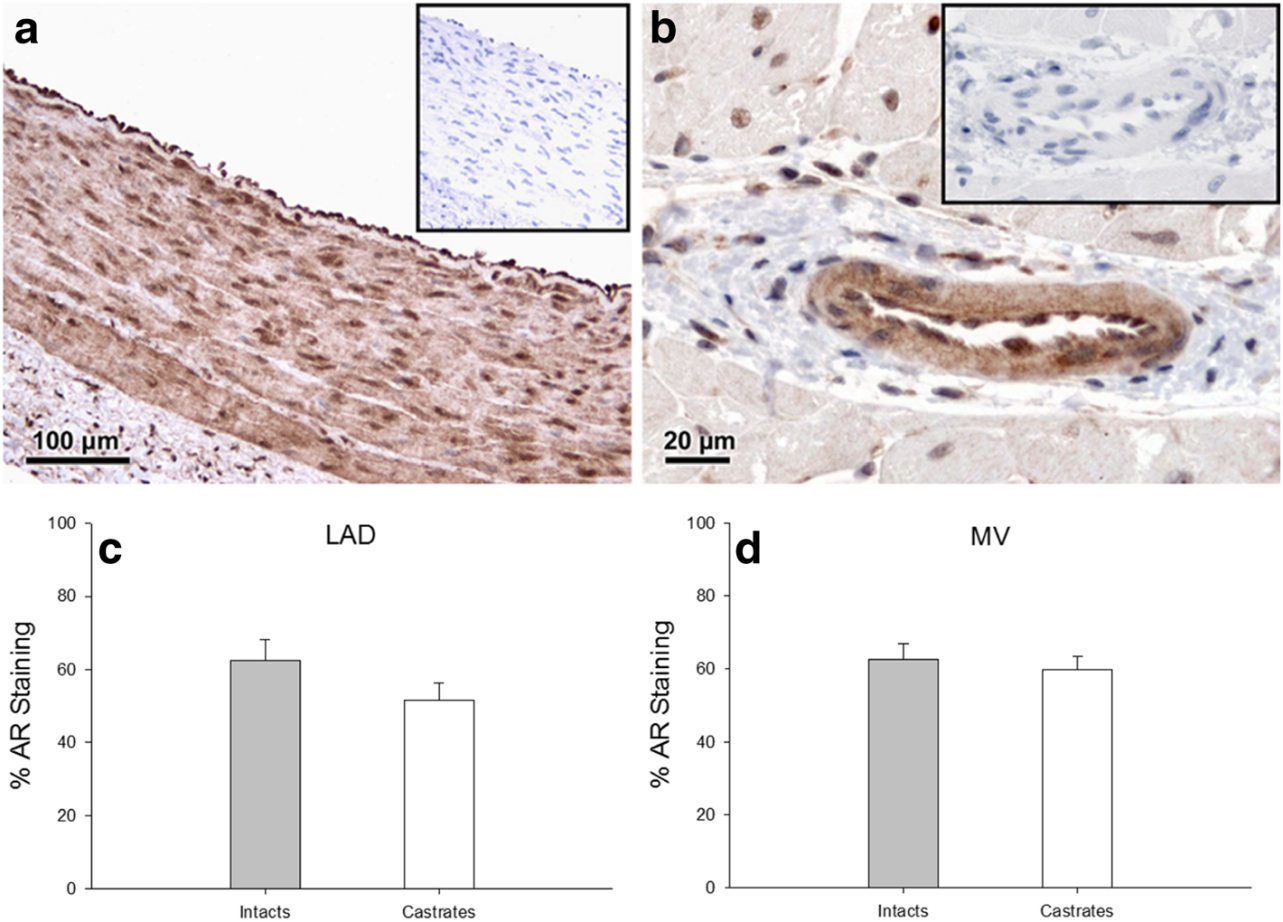
** Androgen receptor immunohistochemistry in intact and castrated males.** Representative photomicrographs demonstrating AR localization in the (**a**) LAD and (**b**) coronary microvessel (MV). Insets: Representative non-immune controls. Quantitative histological analysis of AR staining in the (**c**) LAD (n = 7 for intacts, n = 8 for castrates) and (**d**) left ventricular MV (n = 6 for intacts, n = 5 for castrates). Values are mean ± SEM.

Sections from the LAD demonstrated androgen receptor expression in both the endothelium and in smooth muscle cells of the media. Staining was most intense in the cell nuclei, with cytoplasmic staining also visible (Figure 6A). Microvessels evaluated for androgen receptor expression also demonstrated staining in the endothelium and surrounding smooth muscle cells (Figure 6B). The surrounding adventitia and myocardium demonstrated predominantly nuclear androgen receptor expression. The amount of staining in the intima and media was quantified as a percent of the total vessel area. In the LAD, androgen receptor expression was not statistically different between the intact and castrated males (62.37 ± 5.64 and 51.69 ± 4.56%, respectively) (Figure 6C). In ventricular microvessels, androgen receptor expression was similar in both the intact and castrated males (62.72 ± 4.07 and 59.04 ± 4.83%, respectively)(Figure 6D).

## Discussion

The current study is the first to demonstrate acute *in vivo* vascular responses to exogenous androgen administration are dependent upon endogenous androgen status. The increased coronary conductance in the intact males compared to the castrated males was not associated with differences in androgen receptor expression. Furthermore, these experiments provide the first evidence for a physiologic effect of the androgen, epitestosterone, on the vasculature.

In the present study, nanomolar concentrations of androgen produced statistically significant increases in coronary conductance and microvessel diameter. Circulating testosterone levels in human males range from 11–44 nM [13], though the majority of *in vitro* studies have used supraphysiologic doses in the micromolar range to induce vascular dilation responses [15,16,18,33,34]. In contrast to the majority of *in vitro* data, several *in vivo* studies evaluating the effects of androgen on coronary blood flow have demonstrated significant increases in flow following the infusion of physiologic concentrations of androgen [13,14,17]. The magnitude of the androgen induced flow response, however, has varied in previous studies. Webb *et al.* used 1–100 nM doses of testosterone in men with coronary artery disease to induce a 12-17% increase in coronary blood flow [13], while Chou and colleagues demonstrated a striking 78-86% increase in coronary blood flow following the administration of 1 μM testosterone in anesthetized dogs [14]. In the present study coronary blood flow increased ~5-8% during the testosterone infusions in the anesthetized intact males. The large variation in coronary blood flow responses in the current literature may be related to differences in the methods used to measure or calculate hemodynamic variables, effects of baseline flow on actual concentrations of androgen achieved in the coronary circulation, or perhaps species differences in response to exogenous androgen. A similar study in anesthetized pigs demonstrated increases in coronary blood flow consistent with our data following a 1 μg/L infusion of testosterone [17]. However in contrast to the present study, prepubertal male and female pigs were used for all flow evaluations.

This study is the first to directly compare flow responses between intact and castrated males. During the *in vivo* experiments in the anesthetized pigs, a main effect of castration was observed for the testosterone and 5α-dihydrotestosterone infusions. Conversely, the microvessel studies did not demonstrate statistically significant differences between the dilation responses of intact and castrated subjects to testosterone and 5α-dihydrotestosterone. The reasons for the inconsistencies between the flow and microvessel data may be related to several factors. In the *in vivo* studies, androgen was infused directly into the LAD and therefore had preferential access to the endothelium. However in the microvessel studies, the androgen was administered directly into the bath and as a result contacted the smooth muscle cells of the media first. Because of the different cell types involved and possible differences in drug diffusion through the intima and media, the different experimental conditions create the potential for variable activation of dilator pathways. Another reason for the dilation differences noted in the microvessel studies may be related to the inability of an isolated vessel to mimic the complex, integrated signal conditions present *in vivo*. In the microvessel studies, the effects of androgen on spontaneous tone were evaluated. Conversely, in the *in vivo* studies, numerous other hormones, vasoactive and hemodynamic factors contribute to basal tone [35], and potentially contributed to the differential response between the intact and castrated males. Furthermore, because the *in vivo* studies were performed several hours prior to the microvessel studies, it is possible that androgen administration *in vivo* activated genomic actions in the vasculature that persisted in the *in vitro* studies. These observations indicate that *in vitro* studies are useful to confirm the dilatory action of androgen on the coronary resistance vessels, however in order to determine the mechanism and clinical relevance of androgen induced dilation, *in vivo* studies are critical.

In the current study the role of the androgen receptor in the mechanism of androgen induced vascular dilation was examined. Previous research has evaluated the classical androgen receptor for its potential involvement in vascular dilation. The classic androgen receptor is a nuclear receptor that, upon testosterone or dihydrotestosterone binding, initiates protein synthesis. This genomic pathway; however, typically requires several hours for completion, with some effects detectable at a minimum of 40 minutes [22]. Because the majority of testosterone induced dilation occurs acutely, only several minutes after administration of the hormone, the predominant view is that testosterone must also be involved in a non-genomic pathway. Research has recently identified androgen receptor mediated non-genomic pathways in addition to the classic nuclear androgen receptor pathway. The non-genomic pathways induce rapid responses and involve androgen receptor interactions in the cytoplasm or at the plasma membrane of the cell, leading to the activation of various protein kinases [36]. A recent paper by Yu and colleagues demonstrated the ability of testosterone and 5α-dihydrotestosterone to upregulate the phosphorylation of eNOS in human aortic endothelial cells through androgen receptor mediated activation of the phosphoinositide-3 kinase/Akt pathway. Specifically, the androgen receptor interacted directly with the p85α subunit of PI3-kinase. The upregulation of nitric oxide production in this study was inhibited when the androgen receptor antagonist nilutamide was administered [28]. In the current study, a group of anesthetized, intact male pigs received *in vivo* intracoronary infusions of 5α-dihydrotestosterone and epitestosterone in the presence of flutamide. The dilation induced by epitestosterone was not attenuated in the presence of flutamide; however, the response to 5α-dihydrotestosterone, an androgen with a high affinity for the androgen receptor, was diminished by flutamide. As a caveat, 10 uM flutamide has been used previously in *in vitro* work [16,18], but its efficacy *in vivo* has not been evaluated extensively. It is also possible that androgen causes vascular dilation through multiple pathways, including the direct blockage of L-type Ca^2+^ channels or activation of large-conductance calcium-sensitive K^+^ channels [37]. As a result, only part of the dilation response may be androgen receptor mediated and susceptible to flutamide antagonism. Thus, the results from the present study indicate that the dilation response to 5α-dihydrotestosterone was largely mediated by androgen receptor activation, while the epitestosterone dilation response did not appear to involve activation of the androgen receptor.

Due to the conductance differences between the intact and castrated males in the present study and the potential involvement of the androgen receptor in these responses, androgen receptor expression was evaluated in the conductance and resistance coronary vessels. Previous work has demonstrated an up-regulation of androgen receptor mRNA and protein in vascular smooth muscle cells exposed to varying concentrations of testosterone [38] and reduced androgen receptor staining in castrated males compared to the intact males [32]. We therefore hypothesized that a reduction in circulating testosterone would induce a down regulation of androgen receptor expression and may be associated with the different conductance responses observed in the intact and castrated males. However in the present study androgen receptor mRNA levels and protein in the LAD and coronary microvessels were not different between the intacts and castrates. The current literature regarding post castration androgen receptor expression is indeed quite variable and tissue specific. Prostate epithelial and stromal cells of castrated male guinea pigs demonstrated a lack of androgen receptor staining four days post castration, however brain nuclei staining was unchanged following castration [39]. In rat skeletal muscle, castration reduced androgen receptor expression in the bulbocavernosus muscle, but had no effect on the levator ani muscle [40]; while in bovine skeletal muscle castration was associated with an increase in androgen receptor mRNA in the semitendinosus and triceps brachii but not the splenius [41]. Interestingly, in castrated human males, androgen receptor expression was significantly increased in leukocytes compared to age matched controls [42]. In light of these studies, androgen receptor expression in the vasculature following castration may also be location and cell type dependent. However with regard to the current study, because androgen receptor expression was quantified in both the large conduit vessels and resistance coronary microvessels, it may be concluded that differences in conductance responses between intact and castrated males during exogenous androgen administration were not due to differences in androgen receptor protein expression.

Importantly, the current study is the first to demonstrate the vascular effects of the epimer epitestosterone. Described as a weak anti-androgen, epitestosterone has been thought to be largely biologically inactive. However, coronary conductance was increased in the anesthetized, intact males during the 10 and 100 nM epitestosterone infusions, and in the microvessel experiments, vascular dilation was observed for all doses of epitestosterone. Furthermore, the magnitude of the vasomotor responses was similar to both testosterone and 5α-dihydrotestosterone. Dilation induced by epitestosterone was not attenuated by the presence of flutamide, suggesting that epitestosterone does not function through the androgen receptor to induce vascular dilation. The present study provides the first evidence of a physiologic role for epitestosterone in coronary regulation, and indicates further research is necessary to evaluate its physiological relevance and therapeutic potential.

Several limitations to the current study should be considered. The mean age of the intact males was significantly higher than the castrated males (20.83 ± 4.79 months vs. 3.8 ± 0.39 months). However, post hoc analysis demonstrated the *in vivo* vascular responses in younger intact males (5.33 ± 0.33 months, n = 3) were not different from older intact males (27.60 ± 2.4 months, n = 5), minimizing this concern. The small group size of the conscious animal experiments and the potential effect of anesthesia on the coronary responses in the majority of the flow studies are also acknowledged. However, the conscious, chronically instrumented pigs demonstrated a similar trend for increased coronary conductance during acute androgen administration in the intact compared to the castrated males. With regard to the immunohistochemistry data, while it was demonstrated that differences did not exist in androgen receptor protein expression between the intacts and castrates, the functionality of these receptors is unknown. As a result, it is possible that intracellular signaling pathways are different between the two groups and could contribute to differences in vascular reactivity to androgen. Finally, the serum testosterone levels from all of the pigs used in the flow studies and microvessel studies were not measured, and therefore exact differences in androgen status between the two study groups are unknown. However, historical data from our lab found intact males to have testosterone levels in the range of 200 ng/dL, while castration reduces circulating testosterone by >90% [8].

In conclusion, the present study is the first to examine the role of endogenous hormone status on acute vasomotor responses to physiologic concentrations of testosterone, 5α-dihydrotestosterone and epitestosterone. The data demonstrate significantly increased conductance responses in the intact males compared to the castrates, but this response was not associated with differences in androgen receptor expression as assessed by immunohistochemistry between the two groups. Additionally, the intacts demonstrated a significant dilation response to the epimer epitestosterone, which provides the first evidence for a physiologic role of this androgen on the coronary vasculature. Results from this study suggest that the efficacy of androgen therapy in hypogonadal men may be dependent on endogenous testosterone levels, and further research is necessary to evaluate the dilation pathways involved in acute vascular responses to androgen.

## Competing interests

The authors have no competing interests.

## Author’s contributions

EKO was involved in the inception, design and execution of all experiments and was responsible for data analysis, statistics and contributed to writing the manuscript, including writing the first draft. JRI was involved in all *in vivo* experimental procedures and contributed to writing the manuscript. DKB conceived of the study, and participated in its design and coordination and helped to draft the manuscript. All authors read and approved the final manuscript.
